# Developing questions to assess and measure patients’ perceived survival benefit from adjuvant endocrine therapy in breast cancer: a mixed methods pilot study

**DOI:** 10.1007/s10238-023-01261-4

**Published:** 2024-02-14

**Authors:** Bernard Tawfik, Kendal Jacobson, Ursa Brown-Glaberman, Mikaela Kosich, M. Lee Van Horn, Jacklyn Nemunaitis, Zoneddy Dayao, V. Shane Pankratz, Andrew L. Sussman, Dolores D. Guest

**Affiliations:** 1 University of New Mexico Comprehensive Cancer Center, Albuquerque, NM USA; 2 Department of Internal Medicine, Division of Hematology/Oncology, UNM Comprehensive Cancer Center, 1 University of New Mexico, MSC 07-4025, Albuquerque, NM 87131-0001 USA; 3 University of New Mexico College of Education and Human Sciences, Albuquerque, NM USA; 4 Mexico Department of Internal Medicine, Division of Epidemiology, Biostatistics and Preventive Medicine, University of New, Albuquerque, NM USA; 5 Department of Family and Community Medicine, University of New Mexico, Albuquerque, NM USA

**Keywords:** Breast cancer, Adjuvant endocrine therapy, Patient understanding, Overall survival benefit, PREDICT model

## Abstract

This mixed method study developed multiple question types to understand and measure women’s perceived benefit from adjuvant endocrine therapy. We hypothesis that patients do not understand this benefit and sought to develop the questions needed to test this hypothesis and obtain initial patient estimates. From 8/2022 to 3/2023, qualitative interviews focused on assessing and modifying 9 initial varied question types asking about the overall survival (OS) benefit from adjuvant endocrine therapy. Subsequent focus groups modified and selected the optimal questions. Patients’ self-assessment of their OS benefit was compared to their individualized PREDICT model results. Fifty-three patients completed the survey; 42% Hispanic, 30% rural, and 47% with income < $39,999 per year. Patients reported adequate health care literacy (61.5%) and average confidence about treatment and medication decisions 49.4 (95% CI 24.4–59.5). From the original 9 questions, 3 modified questions were ultimately found to capture patients’ perception of this OS benefit, focusing on graphical and prose styles. Patients estimated an OS benefit of 42% compared to 4.4% calculated from the PREDICT model (*p* < 0.001). In this group with considerable representation from ethnic minority, rural and low-income patients, qualitative data showed that more than one modality of question type was needed to clearly capture patients’ understanding of treatment benefit. Women with breast cancer significantly overestimated their 10-year OS benefit from adjuvant endocrine therapy compared to the PREDICT model.

**Supplementary Information:**

The online version contains supplementary material available at 10.1007/s10238-023-01261-4.

## Introduction

Breast cancer is the most common cancer in the USA, and modern therapies are very effective with cure rate in excess of 80% [1, 2]. Adjuvant endocrine therapy is a mainstay of treatment for the 70% of breast cancer patients whose breast cancer expresses Estrogen Receptors (ER) and/or Progesterone Receptors (PR) but not HER2 [3]. Treatment of curative intent early-stage breast cancer includes surgery, neoadjuvant or adjuvant chemotherapy based on breast tumor receptor status and genomic testing, radiation depending on stage and type of surgery and adjuvant endocrine therapy where an aromatase inhibitor or a selective estrogen receptor modulator is given in a pill form daily for 5–10 years depending on receptor status and individual risk [4] Active research evaluating and recent approvals of additional therapeutic options in the curative intent breast cancer setting in all receptors subtypes include antibody drug conjugates, CDK 4/6 inhibitors and immunotherapy [5–10]. Adjuvant endocrine therapy has been shown to be effective at reducing breast cancer recurrence and improving overall survival (OS) [11]. However, compliance has long been an issue, with only 60% of patients completing the recommended 5 year course of adjuvant endocrine therapy even though higher compliance rates have been shown to improve outcomes including survival [12, 13]. Medication compliance is also worse among Hispanic patients and those living in rural areas [14, 15]. Poor compliance leads to increased recurrence and mortality, especially in those with locally advanced disease [16, 17].

Conversely, over 90% of women with early-stage breast cancer suffer from significant side effects related to adjuvant endocrine therapy and a subset of these women derive only a small benefit [18, 19]. For example, a 65-year-old postmenopausal woman with an estrogen receptor positive, HER2 negative, 1.5 cm breast cancer with no nodal involvement, the 10 year absolute OS benefit from adjuvant endocrine therapy is 0.9% per the PREDICT model [20]. That same example with a 5.5-cm tumor and 4 lymph nodes involved shows a benefit of 6.2%. Women with breast cancer may not understand the relatively small benefit of their therapy on survival, as it is well known that patients do not understand many aspects of their medications in general including the actual benefits and why it is being prescribed [21–23]. It has been previously shown with other breast cancer treatment decisions that patients trust their physicians to provide the best treatment recommendations, so this guidance from a trusted source may discourage patients from discontinuing their adjuvant endocrine therapy despite significant side effects and low benefit [24]. In addition, perceptions of risk has also been shown to be related to behaviors including compliance, thus understanding how women view their risk of breast cancer recurrence and the benefit from adjuvant endocrine therapy at preventing this risk is important for patient decision-making [25]. Additionally, women from ethnic minority populations and those of lower socioeconomic status often have lower health care literacy in general and thus understanding the benefit from adjuvant endocrine therapy is especially important for these women as they have a higher risk of inaccurate perceptions [14, 26].

PREDICT is a validated online tool provided by the National Health Services that estimates absolute OS benefit from therapies including adjuvant endocrine therapy and is widely used [27]. Numerous validated questionnaires exist to assess patient understanding and side effect burden of medication in general but not to assess patient understanding of the perceived benefit from adjuvant endocrine therapy specifically [28]. Data visualization is recognized as an important avenue to assess and disseminate medical information with patient and providers and research for the optimal patient visualization strategy and visuals is ongoing [29, 30].

We hypothesized that women with early-stage breast cancer do not accurately understand the expected OS benefit of their adjuvant endocrine therapy, particularly as estimated by the PREDICT model, and that graphic-based questions would enhance women’s ability to accurately estimate their benefit. In preparation to formally test this hypothesis in a larger future study, we performed this pilot study to create and test questions to enhance accurate measurement of women’s perceived OS benefit from adjuvant endocrine therapy since these do not currently exist. This pilot also provided preliminary data on participants’ perceived survival benefit from adjuvant endocrine therapy.

## Methods

A two-phase exploratory sequential study design was employed with patient surveys followed by semi-structured cognitive interviews with select participants from the sample to include sufficient minority, rural and lower socioeconomic status patients [31]. Similar research designs have been commonly used in oncology research to evaluate side effect burden, quality of life (QOL) and patient-reported outcomes (PRO) [32–38]. This design was chosen to gather baseline data and to elicit concrete suggestions for the optimal question(s) that would accurately measure participants’ perceived survival benefit from adjuvant endocrine therapy which may not have been captured with quantitative surveys alone.

This study enrolled English and Spanish speaking patients at the University of New Mexico Comprehensive Cancer Center from August of 2022 to March of 2023. The University of New Mexico (UNM) Health Sciences Center’s Human Research Protections Office [UNM HRPO] (#19-562) approved the study. The study enrolled women with a history of stage I–III hormone receptor-positive breast cancer who were eligible for any adjuvant endocrine therapy; Tamoxifen, Anastrozole, Letrozole or Exemestane. The women must have initiated, declined, were not recommended, or discontinued the therapy within the last 5 years and be 18 years or older. Patients with ductal carcinoma in situ, men, and women on adjuvant endocrine therapy beyond 5 years were excluded as the benefit beyond 5 years is debated.

### Surveys

Surveys were developed to collect self-reported demographics including race, ethnicity, urban vs rural status, income, education level, age, adjuvant endocrine therapy medication, their provider, and the last time the patient discussed adjuvant endocrine therapy with their provider. Additional survey components included the PROMIS Self-Efficacy for Managing Medications and Treatments questionnaire short form [39, 40] to evaluate patient confidence in choosing, managing and gathering information about medications along with a brief validated questionnaires to assess health literacy by Chew et al. [41, 42]. Surveys were distributed by research coordinators to patients at the time of a regularly scheduled oncology follow-up visit and included informed consent, a study explanation, and the opportunity to decline. The surveys were completed via preloaded tablets using the REDCap platform or paper forms depending on patient preference. Spanish language versions of the survey were available in both formats and were translated by a certified Spanish translator.

### Data extraction

Data extraction done by research coordinators from the Electronic Medical Records (EMR) included age, past medical history, tumor characteristics, type of adjuvant endocrine therapy, additional adjuvant endocrine therapy (if applicable), change or discontinuation reason (if applicable), menopausal status, and PREDICT model results based on that individual patient’s clinical situation.

### Interviews

**Fig. 1:**
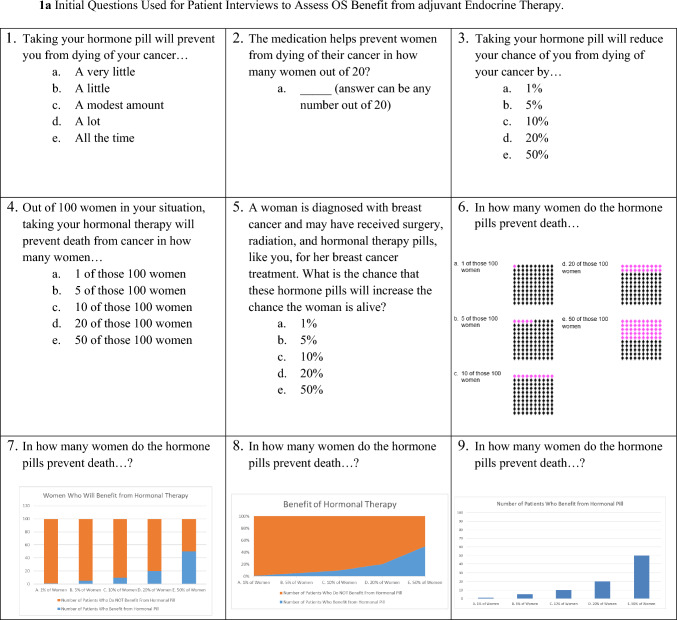

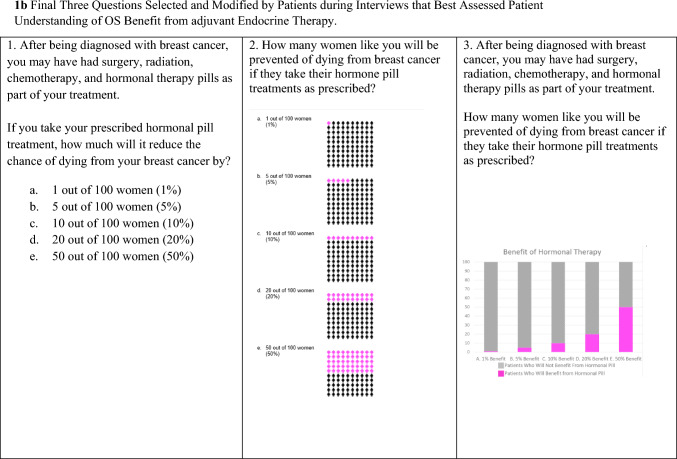
Initial and final questions to measure OS benefit from adjuvant endocrine therapy

**Fig. 2:**
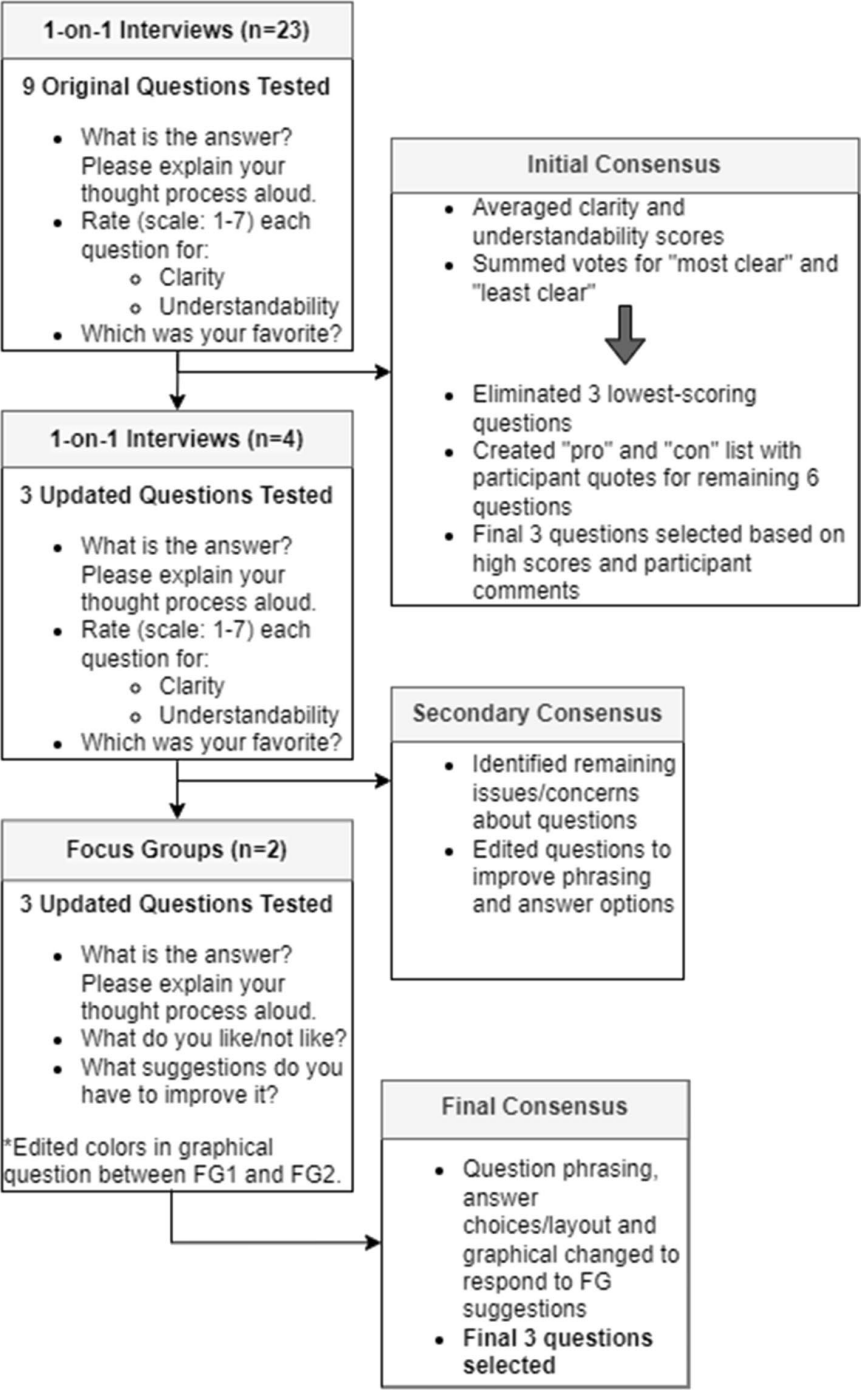
Patient interview and consensus process

At the conclusion of the survey, patients could volunteer to be contacted for a phone or in-person interview. Interviewees were chosen using purposive sampling to represent a mix of rural/urban, Hispanic/Non-Hispanic, and different income levels. The interviews were completed by a Spanish and English bilingual member of the research team who had experience working with the diverse New Mexican population. The goal of the interviews was to select, refine and finalize survey questions designed to assess patient understanding of the benefits of adjuvant endocrine therapy. The nine initial questions we tested were drawn from multiple question styles based on the PREDICT outputs and the COMET study [43] and included prose, fill in the blank, numerical, non-numerical, percentages, iconographs and bar graphs (Fig. 1a). The research team developed semi-structured interview guides based on domains of interest, such as clarity and understandability, adapted from the interview literature [44]. The cognitive piloting occurred in three iterative rounds (Fig. 2) followed by consensus discussion meetings among the study team to determine next steps.

All interviews were conducted via Zoom with the camera on or in person. This allowed the interviewer to identify and respond to visual cues in body language that would suggest hesitation, a positive or negative response to the question, etc., which could then be followed up on with relevant subsequent questions depending on the behavior. The interviewer asked two questions at the start of each interview, to gauge general understanding of the participant’s knowledge and priorities on the difference between cancer recurrence and dying from cancer. The interviewer then showed the test questions one by one. After additionally reading each question to the participant, the interviewer first asked the participant to select the best answer to the question and talk through their thought process aloud. The interviewer then asked the participants to rate the question on two Likert scales from 1 to 7 on clarity and understanding. For example: “How clear was this question for you?” (1 = *not clear at all*, 7 = *totally clear*). Opinions were gathered through probing questions on preference of question and ways to improve test questions for clarity and understanding. The interviewer was experienced in conducting cognitive interviews and did follow-up any verbal or non-verbal reactions including hesitations, spontaneous comments, or expressions such as confusion or uncertainty per published literature [44]. Interviews lasted an average of 45 min. Participants received a $25 gift card for their time. We used feedback from all 27 interviews to select and make edits to the questions.

### Focus groups

We then conducted two focus groups (FG1 and FG2) to assess which question(s) would be the clearest and elicit the most accurate answers. We recruited participants who had already completed a cognitive piloting interview to participate in the focus groups. Focus groups were conducted only in English and via Zoom; they lasted 90 min each and participants were compensated with an additional $25 gift card for their time. Three test questions selected and modified from the previous interviews were the focus of each FG. Two facilitators moderated each discussion and it was explained to the participants that the original nine test questions had been modified or eliminated and why. It was then explained that future changes to the final three questions would be based off participants’ responses in both FGs. Approximately, 30 min were spent discussing each of the three remaining test question. The final few minutes were spent gathering answers to the same overarching questions as in the interviews: “Would anything change if we asked about recurrence instead of dying and what if we asked about increasing chance of survival instead?” Changes suggested by FG1 were discussed during FG2.

### Consensus

As demonstrated in Fig. 2, consensus was an iterative process. Following each round of participant interaction, the study team met to discuss results and participant comments. The *initial consensus* process done during the initial interviews allowed elimination of six questions. The remaining three questions were refined during the *secondary consensus* process during the focus groups. During the *final consensus* following all data collection, the study team made final phrasing and layout changes to the three questions (Fig. 1b). The decision was made to include three questions instead of just one to allow for reproducibility of answers and because many patients seemed to prefer one question type over another but no one question seemed best suited for all participants.

### Endpoints

The primary endpoint of this pilot study was to create questions to accurately and clearly measure women’s perceived OS benefit from adjuvant endocrine therapy to be used in a future study. The secondary endpoint is to measure whether or not women who initiated, declined, were not recommended, or discontinued adjuvant endocrine therapy accurately predicting their OS benefit compared to PREDICT modeling. Other secondary endpoints were to examine health care literacy, understanding of the ability to manage medications and the relationship of the time interval between the last discussions of adjuvant endocrine therapy benefit with accuracy of patients’ predictions.

### Data analysis

The results of the cognitive piloting activities were primarily descriptive in nature. The input provided about each of the various question types from the individuals who engaged in interviews was summarized in tabular form, and counts and percentages were calculated. The percent of participants who provided a positive endorsement of each candidate question type was estimated. We compared a ranking of these positive endorsement proportions across question type. The data from the study surveys and the data obtained from the chart review, were descriptively summarized (means, standard deviations, etc., for quantitatively scaled data, and counts and percentages for categorical data). This included categorizations of the patients’ responses about the likely benefit from adjuvant endocrine therapy. Participants’ ratings of each question’s clarity and understanding were compared, after transformation, using linear mixed effects regression to test for differences in ratings across questions, and to identify those questions with the highest clarity and understanding, while accounting for within-person correlations. Additionally, we combined the ratings of which questions were selected to be the most or least clear into a single perceived clarity score that ranged from positive 100% (equivalent to all respondents selecting the question as being most clear) to negative 100% (equivalent to all respondents selecting the question as being the least clear), and compared these scores across the nine questions using linear mixed effects models. The difference between the patient-assessed likely benefit of treatment with adjuvant endocrine therapy was compared to the PREDICT-estimated likely benefit was assessed using a paired *t*-test. A further assessment of the degree to which breast cancer patients correctly identify the likely benefit of treatment with adjuvant endocrine therapy was obtained by estimating Lin’s concordance correlation coefficient [45]. This correlation coefficient is equal to a value of 1.0 when two ratings result in equal values, and is equal to zero when there is no concordance between the two ratings.

## Results

### Surveys

**Fig. 3:**
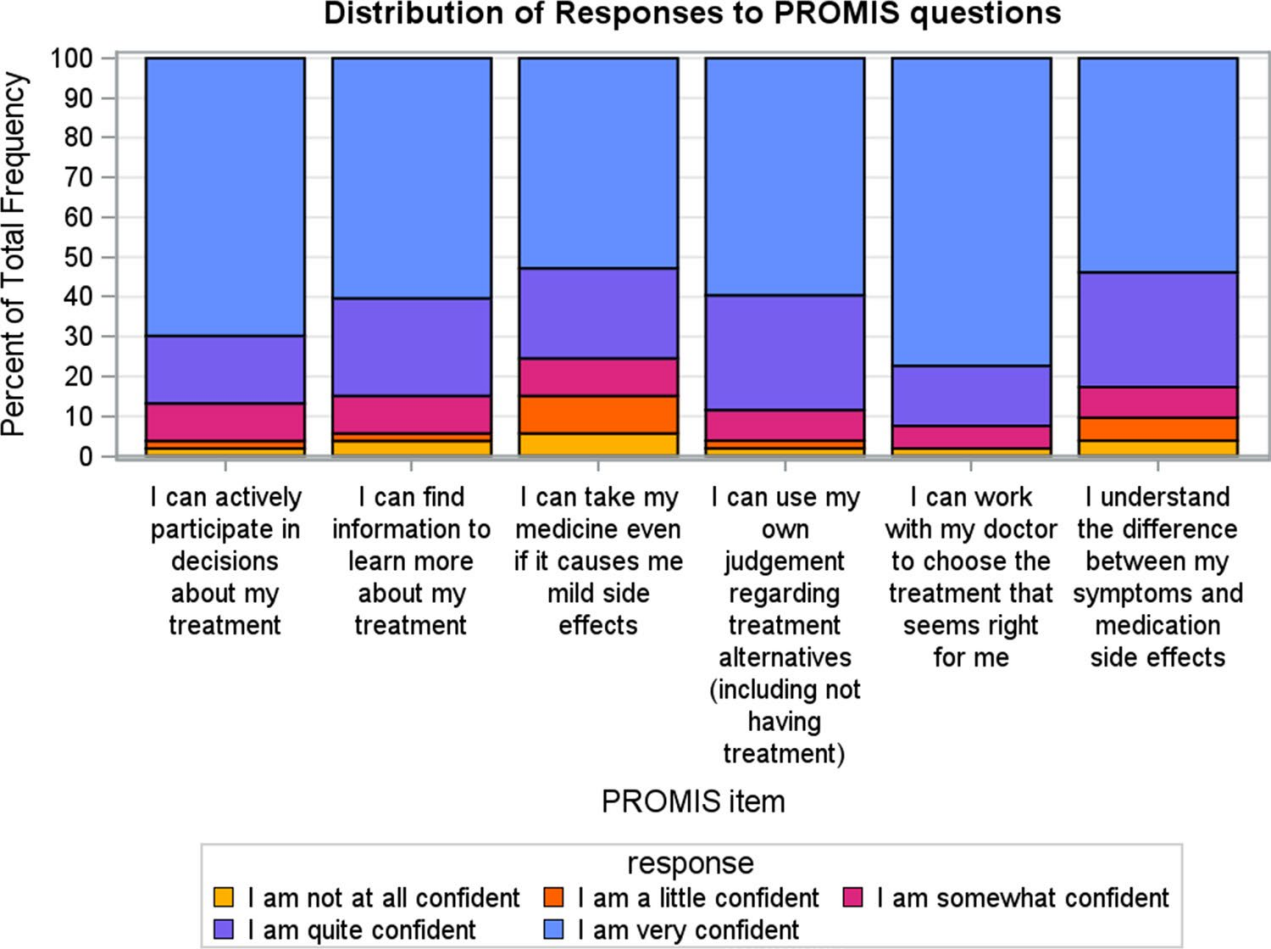
Results of PROMIS

A total of 53 patients completed the initial survey of which 42% were Hispanic, 30% rural, and 47% received $39,999 or less in household income per year (Table 1). Patients answered 88.6% of the questions asked. Patients reported average confidence about treatment and medication decisions with 70% very confident they can actively participate in treatment decisions, 58% very confident they can use their own judgment regarding treatment alternatives or not having treatment with mean score of 49.4 (95% CI 24.4–59.5) compared to a mean of 50 (standard deviation of 10) for US general population (Fig. 3). Additionally, 61.5% of patients reported adequate health care literacy (Supplemental Table 1).

**Table 1:** Patient demographics

Demographics	All		Participated in Interview	
	*N*	%	*N*	%
	53	100	27	50.9
Race and ethnicity				
American Indian or Alaska native	3	5.7	2	7.4
Asian	2	3.8		
Black or African American	1	1.9		
Hispanic/Latino	22	41.5	12	44.4
White	24	45.3	13	48.2
Geographic status				
Rural	16	30.2	9	33.3
Urban	28	52.8	15	55.6
Unsure/Missing	9	17.0	3	11.1
Annual household income				
Less than $10,000–39,999	25	47.2	14	51.9
$40,000–$89,999	16	30.2	5	18.5
$90,000 +	9	17.0	6	22.2
Prefer not to answer/missing	3	5.7	2	7.4
Highest level of education				
Grades 1–8	9	17.0	4	14.8
Some high school	1	1.9		
High school	11	20.8	7	25.9
Bachelor's Degree	14	26.4	8	29.6
Master's Degree	8	15.1	5	18.5
Ph. D or Higher	3	5.7	1	3.7
Trade school	5	9.4	2	7.4
Prefer not to say/missing	2	3.8		
Sex				
Female	53	100	27	100
Age				
30–39 years	4	7.6	1	3.7
40–49 years	6	11.3	5	18.5
50–59 years	14	26.4	6	22.2
60–69 years	13	24.5	5	18.5
70–74 years	14	26.4	10	37.0
80–84 years	2	3.8		
Preferred language to receive healthcare information				
English	40	75.5	19	70.4
Spanish	13	24.5	8	29.6
Insurance type				
No insurance/indignant care	12	22.6	7	25.9
Medicaid	4	7.6	1	3.7
Medicare (with any supplements)	18	34.0	9	33.3
Commercial insurance	16	30.2	8	29.6
Veterans affairs	1	1.9		
Unsure/don't remember/missing	2	3.8	2	7.4
Hormonal therapy				
Received hormonal therapy	44	83.0	21	77.8
Tried hormonal therapy but discontinued	4	7.6	3	11.1
Declined hormonal therapy	5	9.4	3	11.1

### Interviews

Of the 53 patients who completed the initial surveys, 40 (75.5%) indicated willingness to participate in the interview, and 27 (67.5%) interviews were completed. The 13 potential participants who were not interviewed were not reachable or had scheduling conflicts. Demographics were similar for those interviewed with 44% Hispanic, 33% rural, and 47% with $39,999 or less in household income per year (Table 1). The 27 patients ranked all questions from 1 to 7 in terms of clarity and understanding. Linear mixed effects regression suggested that the respondents rated the nine questions differently according to their clarity and understandability (*p* < 0.001). Question #3 was a prose-based question and was the highest rated question with 6.8 (SD = 0.5) for clarity and 6.6 (SD = 0.8) for understanding. The next highest ranked question was #6, which used pictograms and was rated 6.6 (SD = 0.6) for clarity, and 6.6 (SD = 0.5) for understanding. Question #6 (*n* = 14) and Question #7 (*n* = 12) were the most frequently chosen as most clear. Question #7 had a significantly higher clarity score of 37% than those of questions #3, #8, #1, and #2, which had clarity scores of 0, − 11.1, − 14.8, and − 18.5%, respectively. The two questions most frequently chosen as least clear were Question #1 (*n* = 9) and Question #8 (*n* = 8). We found that there were significant differences in perceived clarity among the nine questions (*p* = 0.02). Each of these adjusted mean scores shared the same pooled standard error estimate of 12%. While assessing the questions during these interviews, three larger categories of problems arose: problems with the question wording, problems with the answer choices, and problems with the visual (Table 2).

**Table 2:** Problems with questions and changes made to questions based on interviews

Feedback on…	Sample comments from participants	Changes made
Wording of the question	Question 5: “increase that the chance the woman is alive” it doesn’t seem like a phrase I would hear, it’s less self-explanatory” Question 5: “The last part of the question that I had to read over again—the question itself was a little clunky and I had to read it twice.”	We eliminated the last part of the question, and kept the context from the first part of the question
	Numerous Questions: Many participants found the questions more clear when we added ‘you’ or ‘your’ to the question	Questions were updated with you or your when appropriate
	Numerous Questions: “Maybe when you say hormone treatment, instead say hormone pill treatment—instead of saying therapy you should say hormone pills.”	In the final questions, we used the terms “hormone pill treatments” and “hormonal therapy pills.”
Answer choices	Question 3 and 5: “I don’t like them because of the percentage—people are not going to grasp it—I don’t like the answers.” Question 4: “The question is hard to understand. What does 1 out of 100 mean or does it mean all the people through 100? I don’t understand.”	Added both percentages and n out of 100 to all answer choices Combined question #3, 4, and 5 into new question
	Question 1: “#1 is the least clear, what I call a very little could be different for someone else.” Question 1: “#1 was the worst because it didn’t quantify anything.”	Eliminated question #1
	Question 2: many participants hesitated to answer, or responded with a number out of 20 followed by a percentage that didn’t match. For example, one participant said they thought it was 2 out of 20, and then later said that would be 5%	Eliminated question #2
Graphic elements	Question 8: “I don’t think I understand the continuous graph in the context of this question. You are either benefitting or not. I don’t like this visual presentation of the answer options.”	Eliminated question #8
	Question 7 and 8: Many participants did not like the color of the orange and blue. Some participants noted that the orange really highlighted how many women won’t benefit from treatment	Changed the colors to pink to represent patients who benefit, and grey shading for the patients who don’t
	Question 9: Even though they are both bar graphs, participants preferred question #7 to #9 because it showed contrast by displaying both the patients who benefit from hormonal therapy along with the patients who do not benefit from hormonal therapy	Eliminated question #9

**Fig. 4:**
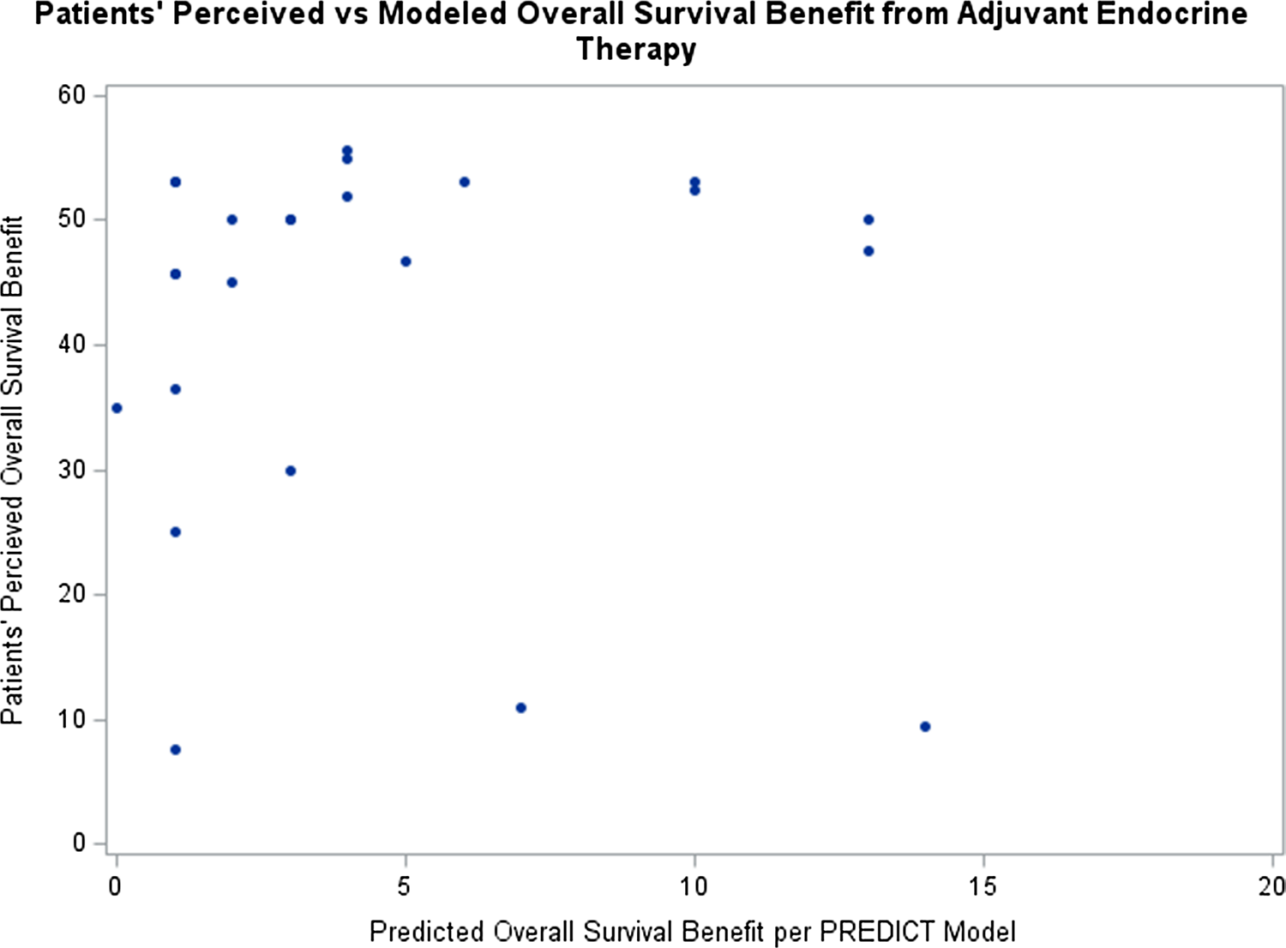
Patients perceived vs. modeled OS benefit from adjuvant endocrine therapy

Each of the questions assessed in this pilot study attempted to prompt participants to estimate the perceived benefit that adjuvant endocrine therapy might provide to them. We compared the resulting self-ratings to the values calculated using the PREDICT model. Across the various question types, the patients estimated a significantly higher average benefit from adjuvant endocrine therapy on survival, with an average benefit reported of 42% (SD = 14.6) when compared to the PREDICT model which showed an average benefit of 4.4% (SD = 4.3) (Fig. 4, *p* < 0.001). Across the question types, the mean patient answer was consistent except for question 1, which was non-numerical prose. For questions 2–9, patients consistently estimated higher survival benefit than the PREDICT model (*p* < 0.001 for all). The most common answers for Question 1 was that adjuvant endocrine therapy prevent people from dying “A modest amount” (22%) and “A lot” (22%). There was no relationship detected between the question answers and when the patient last discussed adjuvant endocrine therapy with their oncologist when comparing 0–6 months vs more than 6 months ago (*p* = 0.22). Comparing the survey averages of the expected treatment benefit to those calculated using the PREDICT tool resulted in an estimate of Lin’s concordance correlation coefficient of − 0.003, indicating essentially no agreement between the breast cancer patients’ ratings and those calculated using PREDICT.

All participants understood the difference between the cancer recurring and dying from cancer. Many participants stated that they thought more about recurrence, because they would not die from cancer unless it recurred first. We also asked participants if they would interpret the questions differently if we asked about recurrence instead of dying. This question was also often followed up by, “what if we asked about increasing survival instead?” Some participants preferred being asked in terms of dying because it was more straight-forward and “didn’t beat around the bush”. Other participants preferred the survival wording because “you see the word death a lot” and because “it gives you hope”.

Patient suggestions for how to improve patients’ understanding of the benefits of adjuvant endocrine therapy included giving participants a brochure or visual aid when they leave, teaching participants more about general medical knowledge, not being too technical, and asking medical team members to be self-aware of the difference between their own risk tolerance and their patient’s risk tolerance when recommending treatment.

## Discussion

The mixed methods study design, featuring patient surveys followed by in-depth qualitative interviews, provided nuanced understandings and preferences of these patients in underrepresented ethnic minority groups, rural and low-income patient populations. The interviews revealed that different patients perceived different questions types as being more or less clear. While most patients preferred a visual representation such as the iconograph (question #6) or bar graph (question #7), a significant minority gave that question type negative feedback and instead preferred a prose format that made the question personal by directly asking about their individual risk reduction (question #3). This suggests that our hypothesis that visual representation would be superior is partially correct and multiple question formats may be needed to ask patients important research questions to ensure clarity for all patients as shown in the three questions that were the final product of this study. Taken a step further, patient facing materials may ideally be represented in multiple formats to increase understanding of the risks and benefits of therapy which are how the PREDICT outputs are currently modeled as these outputs are designed to be shared with patients. This has implications in both the research and clinical settings.

Patients significantly overestimated their 10-year OS benefit from adjuvant endocrine therapy compared to the estimates from the PREDICT model (42 vs 4.4%, *p* < 0.001). Furthermore, the patient-estimated levels of average benefit demonstrated essentially no agreement with the estimates from the PREDICT model. These findings are consistent with our hypothesis that patients do not accurately understand the OS benefit from adjuvant endocrine therapy. These patients reported adequate health care literacy along with average confidence in their medication management and treatment decisions. This adequate confidence regarding their health care literacy and average confidence in medication management is contradicted by the inaccuracy of the patients’ estimates of their OS benefit from adjuvant endocrine therapy. This phenomenon of patients with confidence in their inaccurate predictions is not well described in the healthcare literature and is important for clinical care and a topic for further research. A more general trend of people being overconfident in their decisions is a well-known phenomenon which is likely related [46, 47].

Patients often do not fully understand the associated benefits and side effects of their medications [48, 49]. They often do not fully grasp the risks and benefits of medical interventions which has been well documented in research surrounding consent for surgery and clinical research [50]. Improving questionnaires meant to assess patients symptoms and ask general medical questions are an active area of research that is showing promise with the addition of graphic visualization [51, 52]. Our study adds to this body of evidence showing that women with hormone receptor-positive breast cancer significantly overestimate the OS benefit from adjuvant endocrine therapy. This research also echoes previous findings that visual representation of the benefits of medications are easier to understand for most patients and adds to the literature that some patients find prose more understandable. A combination of these two question types may be the best approach. This is an important area for future research as patients who fully understand the benefits and risks of their medications can make truly informed decisions. Additionally, patients who are confident taking their medications are more likely to be adherent [53, 54]. Since poor adherence is a significant problem with adjuvant endocrine therapy leading to worse outcomes, improvement in adherence could markedly improve patient outcomes. Conversely, adjuvant endocrine therapy has significant side effects that can affect quality of life [55, 56]. Patients who have a low benefit may suffer these side effects without considering discontinuation because they believe adjuvant endocrine therapy to be much more beneficial than it actually is for them. Improving how we assess patient understanding could lead to targeted patient education which could improve outcomes for patients and potentially encourage patients to be more engaged in their health care decisions.

This study holds the potential to illuminate the fact that patients do not understand the benefit of their adjuvant endocrine therapy. If patient do not understand the benefit of their medications, they are less engaged in their care and less adherent to prescribed therapies. Since non adherence to adjuvant endocrine therapy is correlated with worse survival outcomes, improving education that could lead to improved adherence could potentially improve breast cancer survival outcomes. On the opposite end of the spectrum, adjuvant endocrine therapy is known to causes significant side effects. It is very likely that there are women tolerating side effects because they think the predicted benefit is much greater than it is per our models. Education of these women could result in more planned adjuvant endocrine therapy discontinuation, empowering patients to be part of the process and make an informed decision. The problem is that there are significant knowledge gaps in this area as we do not yet have an effective educational intervention for teaching patients about adjuvant endocrine therapy, we do not know if an educational intervention will improve adherence as this research is limited and thus outcomes are not known [57]. Other interventions to improve adherence of adjuvant endocrine therapy have had modest success and thus improving adherence in this patient population is possible and thus worth pursing [58]. Research is needed to develop an easy to implement adjuvant endocrine therapy educational intervention that would improve adherence and outcomes. In the coming years, this could be done with web or smartphone application-based platforms or during already implemented educational components of patient-reported outcomes (PRO) monitoring of side effects to reduce burden on clinical resources. Then, longer trials could be done to assess outcomes measures such as progression-free survival and OS.

This study focused on OS primarily because it is considered the gold standard outcome measure in oncology. However, both OS and recurrence are important to patients as illustrated in the interviews. Similarly, our study does not take into account the significant benefit from adjuvant endocrine therapy as chemoprevention which would decrease the chance of a subsequent cancer in either breast. Patients were aware of the difference between recurrence and OS, suggesting that this can be discussed with patients in the clinic when assessing the benefits of treatment. This is important clinically as many treatments reduce recurrence risk but have not demonstrated improvement in OS and these nuanced risk/benefit conversations can be daunting for patients and providers. The qualitative data from this study would encourage clinicians to have these nuanced conversations as patients are likely to comprehend the difference between recurrence and OS.

This study has inherent limitations. Certain populations such as African American and Asian patients were not well represented in this study, which reduces the generalizability of our findings. This work represents a single academic Comprehensive Cancer Center in one region of the USA with a small number of providers and may not be broadly applicable to other sites, geographical locations or practice types. While a variety of question types for assessing patient understanding of adjuvant endocrine therapy were selected for this study based on the PREDICT model and the COMET study, potentially superior question types may have been missed for inclusion. Patients may have been thinking of and providers may have discussed relative benefit and not absolute benefit which may have led to inaccurate measurements as we measured absolute benefit in this study which we tried to make clear in the survey questions asked and during the interviews. Problems with numeracy may have also led to inaccurate results as many patients of lower socioeconomic and belonging to minority groups struggle to understand mathematical concepts. But graphical representation of questions, having multiple questions asking the same concept and guidance from the interviewer hopefully diminished this effect. Some women who are experiencing side effects may also report a higher benefit because they are in fact reporting the benefit they would need to have to continue to endure the side effects of their adjuvant endocrine therapy as this was documented in two separate interviews. The patient survey data was self-reported and subjective, and therefore may reflect self-reporting biases and/or inaccurate information. This data was not cross referenced against the EMR due to resource constraints. The surveys used were a combination of validated surveys and questions created solely for this study which were not previously piloted, so there may be limitations in reproducibility and validity. In addition, the sample size was relatively small so small differences may not have been detected across sub groups.

In this group of breast cancer patients with substantial representation of ethnic minority, rural and low-income groups, qualitative data showed that more than one modality of question type was needed to clearly capture participants’ perceived survival benefit from adjuvant endocrine therapy including both visual- and prose-based questions. Patients also significantly overestimated their 10-year OS benefit from adjuvant endocrine therapy compared to the PREDICT model. Using this study’s results, these selected and modified questions will be used in a larger study of this patient population to confirm the above findings, examine differences among subgroups, assess providers’ estimate of benefit and providers’ estimate of patient accuracy. Future research should examine educational interventions to improve patient understanding of their adjuvant endocrine therapy, as improved understanding may support a more accurate risk/benefit assessment impacting adherence, informed treatment discontinuation and de-escalation strategies.

## Supplementary Information

Below is the link to the electronic supplementary material.

Supplementary file1 (DOCX 24 kb)

## Data Availability

The datasets generated during and/or analyzed during the current study are not publicly available due to fact that they could be linked to individual patients but are available from the corresponding author on reasonable request.
