# Supplementary material for: Developing questions to assess and measure patients’ perceived survival benefit from adjuvant endocrine therapy in breast cancer: a mixed methods pilot study
Source: Clin Exp Med. 2024 Feb 14;24(1):36. doi: 10.1007/s10238-023-01261-4 (PMC10867096; doi:10.1007/s10238-023-01261-4)
Supplement: Supplementary file 1 — Supplementary file1 (DOCX 24 kb) [file 10238_2023_1261_MOESM1_ESM.docx]

**Supplemental Table 1** Health Literacy Question Results

| **Health Literacy** | **All** | | **Participated in Interview** | |
| --- | --- | --- | --- | --- |
|  |  |  |  |  |
|  | **N** | **%** | **N** | **%** |
| **How often do you have problems learning about your medical condition because of difficulty understanding written information?** |  |  |  |  |
| **Always** | 1 | 1.9% | 1 | 3.7% |
| **Often** | 1 | 1.9% |  |  |
| **Sometimes** | 11 | 20.8% | 2 | 7.4% |
| **Occasionally** | 14 | 26.4% | 9 | 33.3% |
| **Never** | 26 | 49.1% | 15 | 55.6% |
| **How often do you have someone help you read hospital materials?** |  |  |  |  |
| **Always** | 4 | 7.6% | 1 | 3.7% |
| **Often** | 2 | 3.8% | 1 | 3.7% |
| **Sometimes** | 6 | 11.3% | 2 | 7.4% |
| **Occasionally** | 9 | 17.0% | 6 | 22.2% |
| **Never** | 32 | 60.4% | 17 | 63.0% |
| **How confident are you filling out medical forms by yourself?** |  |  |  |  |
| **Extremely** | 30 | 56.6% | 15 | 55.6% |
| **Quite a bit** | 15 | 28.3% | 10 | 37.0% |
| **Somewhat** | 4 | 7.6% | 1 | 3.7% |
| **A little bit** | 2 | 3.8% | 1 | 3.7% |
| **Not at all** | 2 | 3.8% |  |  |
| **Total Health Literacy Score** |  |  |  |  |
| **Limited (3-9)** | 6 | 23.1% | 2 | 7.4% |
| **Marginal (10-12)** | 4 | 15.4% | 6 | 22.2% |
| **Adequate (13-15)** | 16 | 61.5% | 19 | 70.4% |
